# Analysis of family‐wise error rates in statistical parametric mapping using random field theory

**DOI:** 10.1002/hbm.23839

**Published:** 2017-11-01

**Authors:** Guillaume Flandin, Karl J. Friston

**Affiliations:** ^1^ Wellcome Centre for Human Neuroimaging, Institute of Neurology University College London 12 Queen Square, London WC1N 3BG United Kingdom

**Keywords:** family‐wise error rate, statistical parametric mapping, topological inference, random field theory

## Abstract

This technical report revisits the analysis of family‐wise error rates in statistical parametric mapping—using random field theory—reported in (Eklund et al. [2015]: arXiv 1511.01863). Contrary to the understandable spin that these sorts of analyses attract, a review of their results suggests that they endorse the use of parametric assumptions—and random field theory—in the analysis of functional neuroimaging data. We briefly rehearse the advantages parametric analyses offer over nonparametric alternatives and then unpack the implications of (Eklund et al. [2015]: arXiv 1511.01863) for parametric procedures. *Hum Brain Mapp, 40:2052–2054, 2019*. © **2017 The Authors Human Brain Mapping Published by Wiley Periodicals, Inc.**

## INTRODUCTION

Random field theory has been at the heart of statistical parametric mapping in neuroimaging—and its various implementations in academic software—for over two decades. With technical advances in data acquisition, its validity has been revisited every few years to ensure it is fit for purpose [Bennett et al., 2009; Hayasaka and Nichols, 2003; Hayasaka et al., 2004; Nichols, 2012; Pantazis et al., 2005; Woo et al., 2014; Worsley et al., 1996], particularly in relation to controlling family‐wise error. The statistical validity of procedures based on random field theory is important because random field theory offers an efficient and reproducible alternative to nonparametric testing. The advantages of parametric approaches over nonparametric approaches include the following:

- Parametric approaches are more efficient than their nonparametric counterpart by the Neyman–Pearson lemma. This follows because the most efficient test is based on the odds ratio inherent in parametric tests. This means that any nonparametric test can only be as efficient as a parametric test or less efficient.
- Parametric approaches are reproducible. In other words, one obtains the same result when repeating the analysis, unlike the *P*‐values based on samples of the null distribution used in nonparametric tests.
- Parametric approaches eschew the problem of complying with the exchangeability criteria of nonparametric procedures. These criteria make it difficult to apply nonparametric tests to data that have serial correlations or when using hierarchical models.
- Parametric approaches are computationally more efficient because they use distributional assumptions to eschew computationally intensive sampling from a null distribution.

These advantages rest on distributional assumptions that, if violated, render parametric tests inexact. In these instances, one could consider using nonparametric tests. It is, therefore, important to ensure that parametric tests and random field theory are robust to any violations. The analyses reported by [Eklund et al., 2015] speak to this issue. So what conclusions can be drawn from these analyses?

## A REVIEW OF THE EKLUND ET AL. SIMULATION RESULTS

Eklund et al. [2015] assess the family‐wise error rate using parametric and nonparametric tests and a variety of regressors to analyse (publicly available) resting state fMRI data from two sites. They manipulate a number of factors including: (i) inference based on peak height versus spatial extent; (ii) spatial extent inference based on high versus low cluster forming thresholds; (iii) under different levels of spatial smoothing for (iv) block versus event‐related regressors, using (v) one‐ and two‐sample *t*‐tests.

**Figure 1:**
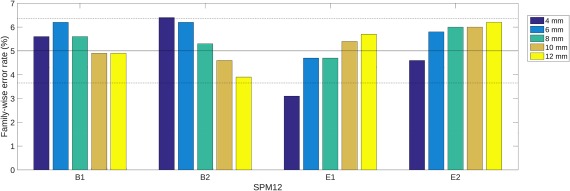
Cluster‐level inference results for a two‐sample *t*‐test (two groups of 10 random subjects, repeated a thousand times) with the Beijing dataset using a cluster forming threshold of *P* = 0.001 (uncorrected) and the SPM12 software (r6685). Five levels of spatial smoothing were evaluated (4, 6, 8, 10 and 12 mm isotropic Gaussian kernels) with four different regressors (see [Eklund et al., 2015] for details).

In brief, they show that parametric inference based on peak height is well‐behaved and provides acceptable family‐wise error control. In contrast, parametric inference based on spatial extent is not valid when, and only when, a low cluster forming threshold is employed. This failure is well known and is consistent with random field theory: the null distribution for spatial extent is based on the Nosko conjecture that provides a distributional form for the spatial extent of a cluster [Friston et al., 1994]. The parameter of this distributional form is fixed using approximations to the expected number of maxima and the total volume above a threshold (see [Flandin and Friston, 2015] for a brief review). Crucially, both the distributional form for the spatial extent and the expected number of maxima (the Euler characteristic) are approximations that are only true in the limit of high thresholds (see fig. 1 in [Friston et al., 1994]). This means that tests based on spatial extent become inexact at low thresholds—as verified numerically by [Eklund et al., 2015].

The effects of smoothing reported in [Eklund et al., 2015] are consistent with random field theory, which assumes a good lattice approximation to a continuous random field. This assumption means that the data have to be smoother than the size of voxels. In other words, increasing the smoothness will lead to more exact inference. Again, this is verified numerically by [Eklund et al., 2015].

The effect of one versus two‐sample *t*‐tests is slightly more difficult to interpret. This is because the authors used the same regressor for all subjects. Arguably, this was a mistake because any systematic fluctuation in resting state timeseries—that correlates with the regressor—will lead to significant one‐sample *t*‐tests against the null hypothesis of zero (e.g., magnetic equilibration effects). This effect is particularly marked for a regressor (called E1) that represents a fast and inefficiently estimated event‐related response every few seconds. Crucially, the nonparametric false positive rates are beyond the 95% confidence intervals. This means that this effect is actually expressed in the data over subjects and, therefore, fails as a model of the null behaviour.

This failure is finessed when comparing parameter estimates between two groups using a two‐sample *t*‐test. In this instance, inferences based on spatial extent fall to acceptable family‐wise error rates. We confirmed this by reproducing the analysis (using the same data and regressors) reported in [Eklund et al., 2015] (see Fig. 1). These analyses use the close to original (3 mm) voxels sizes—as opposed to the upsampled (2 mm voxel) data as analysed in [Eklund et al., 2015].

## CONCLUSION

In summary, we have taken the opportunity to comment on the relative utility of nonparametric and parametric procedures in classical inference: despite the simplicity and robustness of nonparametric tests, there are principled reasons for the predominance of parametric procedures. Having said this, nonparametric tests are extremely useful when validating parametric assumptions—and establishing robustness to their violations. This use of nonparametric tests was exemplified in a recent paper entitled “Cluster failure: Why fMRI inferences for spatial extent have inflated false‐positive rates” [Eklund et al., 2016]. The answer to the question posed by Eklund et al. is that their cluster tests failed because they violated the assumptions underlying analytic (random field theory) approximations to null distributions; in particular, the assumption that clusters are defined by reasonably high thresholds. A useful rule of thumb here is that if clusters have more than one peak, then the cluster forming threshold is probably too low. A sufficiently high threshold is usually guaranteed with the standard cluster forming threshold of *P* = 0.001 (uncorrected). More generally, the simulations reported in Eklund et al speak to the importance of revisiting the robustness of statistical tests as the nature of imaging data evolves and new researchers—who are not familiar with these foundational issues—enter the field.
